# Biological Function of Long Non-coding RNA (LncRNA) Xist

**DOI:** 10.3389/fcell.2021.645647

**Published:** 2021-06-10

**Authors:** Wenlun Wang, Lu Min, Xinyuan Qiu, Xiaomin Wu, Chuanyang Liu, Jiaxin Ma, Dongyi Zhang, Lingyun Zhu

**Affiliations:** Department of Biology and Chemistry, College of Liberal Arts and Sciences, National University of Defense Technology, Changsha, China

**Keywords:** long non-coding RNA, lncRNA Xist, cancer, disease, X-chromosome inactivation, X-chromosome inactivation center

## Abstract

Long non-coding RNAs (lncRNAs) regulate gene expression in a variety of ways at epigenetic, chromatin remodeling, transcriptional, and translational levels. Accumulating evidence suggests that lncRNA X-inactive specific transcript (lncRNA Xist) serves as an important regulator of cell growth and development. Despites its original roles in X-chromosome dosage compensation, lncRNA Xist also participates in the development of tumor and other human diseases by functioning as a competing endogenous RNA (ceRNA). In this review, we comprehensively summarized recent progress in understanding the cellular functions of lncRNA Xist in mammalian cells and discussed current knowledge regarding the ceRNA network of lncRNA Xist in various diseases. Long non-coding RNAs (lncRNAs) are transcripts that are more than 200 nt in length and without an apparent protein-coding capacity (Furlan and Rougeulle, 2016; Maduro et al., 2016). These RNAs are believed to be transcribed by the approximately 98–99% non-coding regions of the human genome (Derrien et al., 2012; Fu, 2014; Montalbano et al., 2017; Slack and Chinnaiyan, 2019), as well as a large variety of genomic regions, such as exonic, tronic, and intergenic regions. Hence, lncRNAs are also divided into eight categories: Intergenic lncRNAs, Intronic lncRNAs, Enhancer lncRNAs, Promoter lncRNAs, Natural antisense/sense lncRNAs, Small nucleolar RNA-ended lncRNAs (sno-lncRNAs), Bidirectional lncRNAs, and non-poly(A) lncRNAs (Ma et al., 2013; Devaux et al., 2015; St Laurent et al., 2015; Chen, 2016; Quinn and Chang, 2016; Richard and Eichhorn, 2018; Connerty et al., 2020). A range of evidence has suggested that lncRNAs function as key regulators in crucial cellular functions, including proliferation, differentiation, apoptosis, migration, and invasion, by regulating the expression level of target genes via epigenomic, transcriptional, or post-transcriptional approaches (Cao et al., 2018). Moreover, lncRNAs detected in body fluids were also believed to serve as potential biomarkers for the diagnosis, prognosis, and monitoring of disease progression, and act as novel and potential drug targets for therapeutic exploitation in human disease (Jiang W. et al., 2018; Zhou et al., 2019a). Long non-coding RNA X-inactive specific transcript (lncRNA Xist) are a set of 15,000–20,000 nt sequences localized in the X chromosome inactivation center (XIC) of chromosome Xq13.2 (Brown et al., 1992; Debrand et al., 1998; Kay, 1998; Lee et al., 2013; da Rocha and Heard, 2017; Yang Z. et al., 2018; Brockdorff, 2019). Previous studies have indicated that lncRNA Xist regulate X chromosome inactivation (XCI), resulting in the inheritable silencing of one of the X-chromosomes during female cell development. Also, it serves a vital regulatory function in the whole spectrum of human disease (notably cancer) and can be used as a novel diagnostic and prognostic biomarker and as a potential therapeutic target for human disease in the clinic (Liu et al., 2018b; Deng et al., 2019; Dinescu et al., 2019; Mutzel and Schulz, 2020; Patrat et al., 2020; Wang et al., 2020a). In particular, lncRNA Xist have been demonstrated to be involved in the development of multiple types of tumors including brain tumor, Leukemia, lung cancer, breast cancer, and liver cancer, with the prominent examples outlined in Table 1. It was also believed that lncRNA Xist (Chaligne and Heard, 2014; Yang Z. et al., 2018) contributed to other diseases, such as pulmonary fibrosis, inflammation, neuropathic pain, cardiomyocyte hypertrophy, and osteoarthritis chondrocytes, and more specific details can be found in Table 2. This review summarizes the current knowledge on the regulatory mechanisms of lncRNA Xist on both chromosome dosage compensation and pathogenesis (especially cancer) processes, with a focus on the regulatory network of lncRNA Xist in human disease.

## The Role of LncRNA Xist in X Chromosome Dosage Compensation

In most mammals, sex is determined by a system based on X and Y chromosomes (Deng et al., 2014), with males holding the XY chromosome and females XX. Dosage compensation is thus needed to ensure equivalent expression levels of sex-linked and autosomal genes (Polito et al., 1990; Bone and Kuroda, 1996; Larsson and Meller, 2006; Disteche, 2012, 2016; Ferrari et al., 2014) despite the presence of an extra X-chromosome in female cells (Deng et al., 2014). X-chromosome inactivation (XCI), which refers to the random selection and transcriptional silence of one of two X-chromosomes in females at the early stages of embryonic development, is a unique dosage compensation mechanism in mammals (Waldron, 2016; Bar et al., 2019; Strehle and Guttman, 2020; Yu B. et al., 2020). In most placental mammals, there are two waves of XCI: the imprinted XCI exists in the fertilized embryo and extraembryonic tissues, and the random XCI persists in the inner cell mass (after implantation around embryonic day 5.5), yet humans lack the imprinted XCI and instead have X chromosome dampening (XCD) (Ropers et al., 1978; Harper et al., 1982; Kung et al., 2013; Lee et al., 2013; van Bemmel et al., 2016; Finestra and Gribnau, 2017; Sahakyan et al., 2018).

XCI is subdivided into distinct phases: initiation, establishment, and maintenance of the inactive X-chromosome (Gontan et al., 2011; Maduro et al., 2016). Initiation phase is a stochastic process (Spatz et al., 2004; Maduro et al., 2016; Jegu et al., 2017) that involves X-X pairing, counting, and XCI activation (xist activation, etc.) processes, and ensures that any number of X chromosomes randomly generate only one active X chromosome (Xa) expressed in each female cell and inactive X chromosome (Xi) is hetero-chromatinized and silenced in female cells. Establishment phase (Spatz et al., 2004; Maduro et al., 2016; Colognori et al., 2020) involves building a chromosomal memory that persists through the ensuring maintenance phase and ensures stable retention of repressive heterochromatin. Once the establishment phase is completed, the XCI is remarkably stable and becomes more difficult to reactivate. Maintenance phase is keeping the silenced state of XCI after the establishment phase via continuing lncRNA Xist expression. Once Xi is established, the Xi fully maintains its silent configuration and is clonally propagated throughout cell divisions (Maduro et al., 2016; Finestra and Gribnau, 2017). Numorous studies suggest that all three phases of XCI are governed by the lncRNA Xist (Lu et al., 2017; Sahakyan et al., 2018; Sidorenko et al., 2019).

**FIGURE 1:**
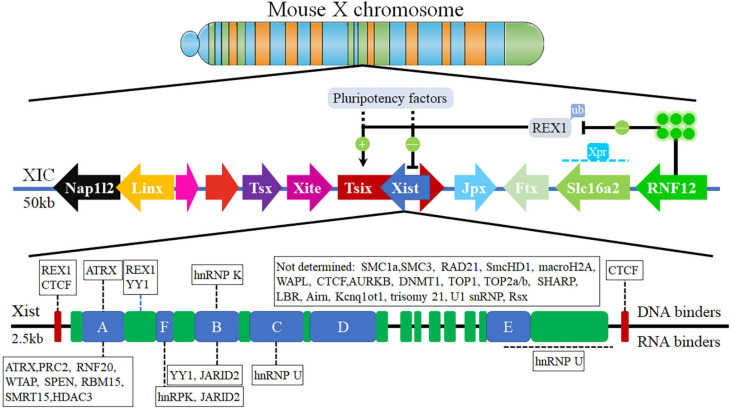
The X-chromosome Inactivation Center (Maduro et al., 2016). The X inactivation center consists of the different genes located and multiple genes encoding lncRNA, containing Xist, Tsix, Tsx, Xite, Jpx, Ftx, DNA binders, and RNA binders.

XIC is the X-linked minimal genetic region which contains various factors and genes, such as Xist and Tsix, that are necessary and sufficient to initiate the XCI process in female cells (Willard, 1996; Sherstyuk et al., 2013; Hwang et al., 2015; Loda and Heard, 2019). XIC (Figure 1) is located in 100–500 kb region of mouse X chromosome and 2.3 Mb syntenic region of human X chromosome, and includes a cluster of lncRNA loci, such as Ftx, Jpx, Xist, Tsix, Xite, RepA, and so on (Spatz et al., 2004; Augui et al., 2011; Maduro et al., 2016; Jegu et al., 2017; Loda and Heard, 2019; Sidorenko et al., 2019). lncRNA Xist exists inside XIC, specifically at a location 15 kb downstream from Tsix antisense (Sado and Brockdorff, 2013; Gendrel and Heard, 2014; Mira-Bontenbal and Gribnau, 2016; da Rocha and Heard, 2017; Pintacuda et al., 2017b; Monfort and Wutz, 2020), and contains several functional domains that are a series of conservation repetitive motifs of A-to-F repeats (Figure 1). lncRNA Xist is transcriptionally activated with the initiation of the XIC process and is also believed to contribute to the complete process of XCI as a master regulator.

**FIGURE 2:**
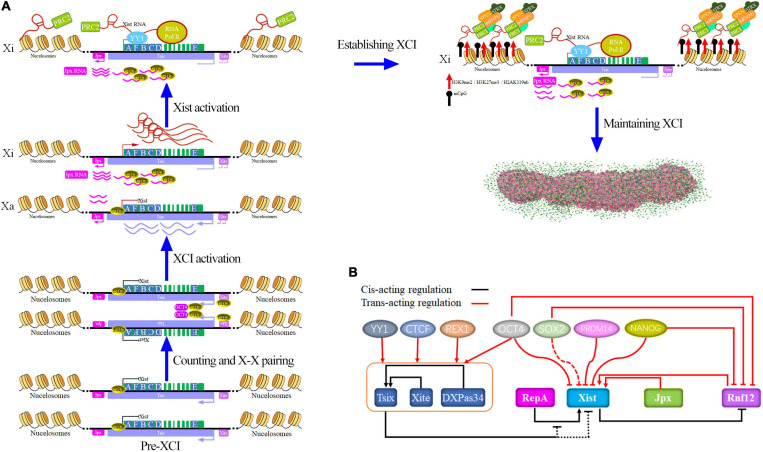
Model for Xist and Xist regulation at the process of XCI. **(A)** The process of dynamic and multifaceted modulation of XCI by lncRNA Xist. lncRNA Xist is a multitasking RNA that recruits protein complexes (such as OCT4, CTCF, Tsix, Xite, PRC1, PRC2, SPEN, ATRX, hnRNPU, hnRNPK, SHARP, HDAC3, LBR, Airn, Kcnq1ot1, RBM15, WTAP, trisomy 21, U1 snRNP, Rsx, Sox2, PRDM14, Jpx, Rnf12, and RepA) to initiate, establish, and maintain the XCI state by histone modifications, DNA methylation, and H4 hypoacetylation. **(B)** LncRNA Xist regulation network of genetic interactions (Augui et al., 2011). Note that here arrows do not necessarily imply direct regulation.

lncRNA Xist and its associated chromatin modifying complex plays a vital role in the regulation of the XCI process (Figure 2). A detailed description of the XCI process is beyond the scope of this review, and more specific detail is given in references (Spatz et al., 2004; Augui et al., 2011; Jegu et al., 2017), We briefly described regulatory process involved in LncRNA Xist in the review (Figure 2). During the initiation phase, the complex factors (OCT4, CTCF, Tsix, Xite, etc.), which separately bind the Xa and Xi, facilitates the X chromosome pairing and counting in the embryo after fertilization (Xu et al., 2006; Donohoe et al., 2009; Kung et al., 2015). After counting and pairing, XCI initiation is also accompanied by Tsix, Xist, etc. upregulation which is controlled by the network of genetic interactions (Figure 2B), such as Tsix, Sox2, PRDM14, OCT4, Jpx, Rnf12, and RepA (Augui et al., 2011; Khamlichi and Feil, 2018). When complete onset of XCI occurs, they employ divergent transcription fates with one becoming the Xa chromosome and the other becoming the Xi chromosome (Jegu et al., 2017). In Xi, lncRNA Xist activation and expression is modulated by numerous factors, such as pluripotency factor (NANOG, OCT4, SOX2, PRDM14, and REX1), RNF12, Tsix, and RepA (PcG protein recruitment), and more information is given in reference (Augui et al., 2011; Khamlichi and Feil, 2018). The regulation of Tsix expression is beyond the scope of this review, and more specific details can be found in references (Willard, 1996; Gontan et al., 2012; Gayen et al., 2015). Once Xist expression has been activated, Xist binds Polycomb repressive complex 2 (PRC2) via Repeat A formed Xist-PRC2 complex, and YY1 tethers the PRC2-Xist complex through Repeat C to the Xi nucleation center which obtains lncRNA Xist-PRC2 complex by the RNA polymerase II (RNA Pol II) (Jeon and Lee, 2011; Thorvaldsen et al., 2011; Makhlouf et al., 2014; Chigi et al., 2017).

After the initiation phase, LncRNA Xist recruits protein complex factors excluding RNA Pol II, and induces a global suppression of lncRNA Xist topologically associated domains (TAD), which is involved in epigenetic modification and chromatin compaction to the Xi chromosome to spreads along the Xi at the established phase (Giorgetti et al., 2016; Mira-Bontenbal and Gribnau, 2016; Finestra and Gribnau, 2017; van Bemmel et al., 2019; Galupa et al., 2020). These protein complexes (Figure 2A) include the heterogeneous nuclear protein U (hnRNPU; also known as SAF-A), which is required for lncRNA Xist localization (Hasegawa et al., 2010; Kolpa et al., 2016; Sakaguchi et al., 2016; Loda and Heard, 2019), heterogeneous nuclear ribonucleoprotein K (hnRNPK), which is required for Xist-mediated chromatin modifications and Polycomb recruitment but not lncRNA Xist localization (Chu et al., 2015; Pintacuda et al., 2017a; Loda and Heard, 2019; Wang et al., 2019g), and the gene-silencing factor Spen, which is not required for Xist RNA localization (Chu et al., 2015; Monfort et al., 2015; Loda and Heard, 2019; Dossin et al., 2020) and binds to C, B, F, and A repeats at the 5′ end of the lncRNA Xist. ATRX directs binding to two major Polycomb repressive complexes (PRCs). -PRC1 and -PRC2 are involved in epigenetic silencing (acetylation of histone H3 and H4 and CpG island methylation, etc.) (Sarma et al., 2014; Minajigi et al., 2015; Pinheiro and Heard, 2017; Colognori et al., 2019; Lee et al., 2019; Wang et al., 2019a; Chen and Zhang, 2020). Other protein complexes (Mira-Bontenbal and Gribnau, 2016; Loda and Heard, 2019) also take part in the lncRNA Xist spreading procession, such as SHARP (McHugh et al., 2015), HDAC3 (Zylicz et al., 2019), LBR (Chen C.K. et al., 2016; Nesterova et al., 2019), Airn and Kcnq1ot1 (Schertzer et al., 2019), RBM15 and WTAP (Mira-Bontenbal and Gribnau, 2016), trisomy 21 (Jiang et al., 2013), U1 snRNP (Yin et al., 2020), Rsx (Grant et al., 2012), and CdK8 (Postlmayr et al., 2020). LncRNA Xist recruits repressive complexes, which leads to immediate histone modifications and DNA methylation (such as H2AK119Ub, H3K27me3, and CpG island) and coats on the Xi to build Xi (Mira-Bontenbal and Gribnau, 2016; Pinheiro and Heard, 2017; Wang et al., 2020a). Taken together, the Xi has been established and maintained in an inactive state by continuous synthesis of lncRNA Xist RNA.

## The Role of LncRNA Xist in Cancer

Cancer, of which there are over 200 different types, is a complex disease in which cells in a specific tissue are no longer fully responsive to the signals within the tissue that regulate cellular differentiation, survival, proliferation, and death. As a result, these cells accumulate within the tissue, causing local damage and inflammation Cancer cells proliferate (growth) out of control, spread to other tissues (metastasize), and lose the ability to die via the normal process of cell apoptosis (death). The discovery of lncRNA Xist has contributed to cancer development and progression by regulation of the downstream signaling processes (Table 1). This also provides a window into the understanding of aberrant expression of lncRNA Xist associated with tumorigenesis, metastasis, and tumor stage. lncRNA Xist is a novel potential biomarker and potentially could be used in diagnosis and therapy for different types of cancer.

**TABLE 1:** LncRNA Xist and miRNA in cancer.

**Cancer type**	**miRNA**	**Target**	**Mechanism of action and function**	**Signaling pathway**	**References**
Bladder cancer	miR-124, miR-139-5p, miR-200c, miR-133a, miR-335	AR, Wnt1, TET1, p53	Aberrant expression lncRNA Xist is involved in cancer cells growth, proliferation, metastasis, migration, invasion, apoptosis, epithelial mesenchymal transition and drug resistance	TGF-beta signaling pathway, PIK3/AKT signaling pathway, mTOR signaling pathway, Wnt/ β-catenin signaling pathway, p53 signaling pathway, MAPK signaling pathway, FOXO signaling pathway, HIF-1 signaling pathway, Thyroid hormone signaling pathway, Notch signaling pathway, C-type lectin receptor signaling pathway, JAK-STAT signaling pathway, AGE-RAGE signaling pathway, Pathways of neurodegeneration—multiple diseases and ECM-receptor interaction, etc.	Hu et al., 2017; Xiong et al., 2017; Xu R. et al., 2018; Hu B. et al., 2019; Zhou et al., 2019c; Chen D. et al., 2020
Breast cancer	miR-155, miR-20a, miR-200c-3p, miR-454, miR-92b, miR-503, miR-125b-5p, miR-362-5p	CDX1, TP53, ANLN, Slug, ESA, PHLPP1, AKT, MSN, cMet, NLRC5, UBAP1			Xing et al., 2018; Zhao L. et al., 2018; Zheng R. et al., 2018; Li et al., 2020c,d,e; Liu et al., 2020a; Zhang et al., 2020a
Colorectal cancer	miR-137, miR-132-3p, miR-486-5p, miR-93-5p, miR-124, miR-30a-5p, miR-338-3p	EZH2, MAPK1, NRP-2, HIF1A, AXL, METTL14, SGK1, ROR1, PAX5			Chen D.L. et al., 2017; Song et al., 2017; Liu et al., 2018a, 2019a; Zhu J. et al., 2018; Zhang et al., 2019d; Ma et al., 2020; Yang L.G. et al., 2020
Glioblastoma	miR-152, miR-27a, miR-429, miR-137, miR-126, miR-133a, miR-29c, miR-204-5p	Smurf1, ZO-2, FOXC1, Rac1, SLC1A5, IRS1, SOX4, MMR, Bcl-2, ASCT2			Du P. et al., 2017; Wang Z. et al., 2017; Yu H. et al., 2017; Cheng Z.H. et al., 2020; Luo C.X. et al., 2020; Shen J. et al., 2020; Sun Y. et al., 2020; Yao et al., 2020; Zhao Q. et al., 2020
Hepatocellular carcinoma	miR-29b, miR-92b, miR-155-5p, miR-200b-3p, miR-139-5p, miR-194-5p, miR-497-5p, miR-181a	HMGB1, SMAD7, SOx6, PTEN, PDK1, AKT, MAPK1, PDCD4, PTEN			Zhuang et al., 2016; Chang et al., 2017; Mo et al., 2017; Kong et al., 2018; Lin et al., 2018; Liu and Xu, 2019; Xie et al., 2019; Zhang et al., 2019h
Nasopharyngeal	miR-34a-5p, miR-29c, miR-491-5p, miR-148a-3p, miR-381-3p	E2F3, Notch3, ADAM17, NEK5, PDCD4, Fas-L			Song et al., 2016; Han et al., 2017; Cheng Q. et al., 2018; Shi et al., 2020; Zhao C.H. et al., 2020
Lung cancer	miR-140, miR-363-3p, let-7i, miR-449a, miR-374a, miR-212-3p, miR-186-5p, miR-137, miR-744, miR-367, miR-141, miR-16, miR-335, miR-144-3p, miR-17, miR-142-5p	iASPP, TCF-4, MDM2, BAG-1, HIF1A-AS1, KLF2, Bcl-2, LARP1, CBLL1, PXN, Notch-1, RING1, ZEB2, CDK8, SOD2, ROS, SMAD2, p53, NLRP3, MDR1, MRP1, ATG7, PAX6			Sun J. et al., 2017; Sun W. et al., 2017; Wang H.Y. et al., 2017; Xu Z.Z. et al., 2017; Zhang Y.L. et al., 2017; Jiang H.J. et al., 2018; Li C. et al., 2018; Wang et al., 2018c, 2019c; Liu et al., 2019b; Qiu et al., 2019; Tian et al., 2019; Zhou et al., 2019e; Jiang et al., 2020; Rong et al., 2020; Xu et al., 2020
Osteosarcoma	miR-21-5p, miR-193-3p, miR-195-5p, miR-137, miR-302b, miR-375-3p, miR-153	p21, NF-kB, PUMA, PDCD4, RSf1, YAP, RAP2B, AKT, mTOR, SNAl1			Wu D.P. et al., 2017; Zhang and Xia, 2017; Lv et al., 2018; Yang C. et al., 2018; Li H. et al., 2019; Sun X. et al., 2019; Wen et al., 2020
Pancreatic cancer	miR-133a, miR-140, miR-124, miR-34a-5p, miR-34a, miR-141-3p, miR-429	EGFR, iASPP, YAP, EGFR, TGF-β2, ZEB1			Liang et al., 2017; Wei W. et al., 2017; Sun Z. et al., 2018; Shen et al., 2019; Sun and Zhang, 2019; Zou et al., 2020
Retinoblastoma	miR-21-5p, miR-124, miR-101, miR-140-5p, miR-200a-3p	VEGF, NKILA, STAT3, ZEB1, ZEB2, SOX4, NRP1			Hu et al., 2018; Cheng Y. et al., 2019; Dong et al., 2020; Wang et al., 2020c; Zhao H. et al., 2020
Cervical cancer	miR-200a, miR-140-5p, miR-889-3p	Fus, ORC1, SIX1			Zhu H. et al., 2018; Chen X. et al., 2019c; Liu et al., 2020d
Gastric cancer	miR-101, miR-497, miR-185, miR-337	EZH2, MACC1, TGF-β1, MDR1, MRP1, JAK2			Chen D.L. et al., 2016; Ma L. et al., 2017; Zhang Q. et al., 2018; Zheng W. et al., 2020
Melanoma	miR-21, miR-139-5p, miR-217	PI3KR1, ROCK1			Pan et al., 2019; Zhang et al., 2019f; Tian K. et al., 2020
Esophageal cancer	miR-101, miR-494	EZH2, CDK6			Wu X. et al., 2017; Chen et al., 2019f
Laryngeal squamous cell carcinoma	miR-124-3p, miR-144, miR-125-5p	EZH2, IRS1, TRIB2			Xiao D. et al., 2019; Cui et al., 2020; Liu et al., 2020b
Ovarian cancer	miR-214-3p, miR-150-5p	PTEN, PDCD4			Zuo et al., 2019; Wang and Li, 2020
Neuroendocrine tumor	miR-424-5p	bFGF			Zhou et al., 2019b
Neuroblastoma	miR-375	EZH2, DKK1, L1CAM			Yang H. et al., 2020
Thyroid cancer	miR-34a, miR-141	MET			Liu et al., 2018c; Xu Y. et al., 2018
Colon cancer	miR-34a	Wnt/β-catenin			Sun N.N. et al., 2018
Renal cell carcinoma	miR-106b-5p, miR-302c	p21, SDC1			Zhang J. et al., 2017; Sun K. et al., 2019
Prostate cancer	miR-23a	RKIP, LINE-1			Laner et al., 2005; Du Y. et al., 2017
Chordoma	miR-124-3p	iASPP			Hai, 2020

### LncRNA Xist in Bladder Cancer

Bladder cancer is more common in men than in women, with respective incidence and mortality rates of 9.6 and 3.2 per 100,000 in men, which is about 4 times that of women globally (Chen W. et al., 2016; Bray et al., 2018). lncRNA Xist has recently been reported to regulate bladder cancer development through regulating several miRNAs or other target genes. lncRNA Xist exerts an oncogenic role through binding to miR-124, miR-139-5p, miR-200c, miR-133a, and miR-335 targets AR, Wnt1, TET1, and p53, which affect cell growth, invasion and migration, and metastasis (Hu et al., 2017; Xiong et al., 2017; Xu R. et al., 2018; Hu B. et al., 2019; Zhou et al., 2019c; Chen D. et al., 2020). This research uncovered that lncRNA Xist may be invoked as a potential therapeutic and prognostic biomarker for bladder cancer.

### LncRNA Xist in Breast Cancer

Breast cancer accounts for almost one in four cancer cases among women, with respective incidence and mortality rates of 24.2 and 15.0%, and is the most commonly diagnosed cancer and leading cause of cancer death in women globally (Chen W. et al., 2016; Bray et al., 2018). Some previous studies have suggested that deregulation of lncRNA Xist plays a vital role in the pathogenesis of both inherited and sporadic breast cancer (Kawakami et al., 2004; Soudyab et al., 2016). The Breast Cancer 1 protein (BRCA1) is a tumor suppressor. Reduced expression of BRCA1 leads to increased risk of breast cancer development (Romagnolo et al., 2015). LncRNA Xist, which is dependent on the production of BRCA1 and may participate in regulating breast cancer development, is highly expressed in BRCA1-like breast cancer as a predictive biomarker (Sirchia et al., 2005, 2009; Vincent-Salomon et al., 2007; Schouten et al., 2016). It is thought that histone modifications (histone deacetylase inhibitor) and DNA methylation plays a critical role in breast cancer growth and metastasis (Librizzi et al., 2015; Shukla et al., 2019). Some research indicated that breast tumors frequently display major epigenetic instability of XI which is mediated by lncRNA Xist, and this phenomenon regulates breast cancer cells’ proliferation and differentiation (Salvador et al., 2013; Chaligne et al., 2015). In addition to the indirect regulation of competing endogenous RNA (ceRNAs), studies published to date have demonstrated that knockdown or overexpressed LncRNA Xist in breast cancer results in sponging five miRNAs, containing miR-155, miR-20a, miR-200c-3p, miR-125b-5p, and miR-362-5p, and positively regulates the downstream targets including CDX1, TP53, ANLN, NLRC5, and UBAP1, which affects breast cancer cells’ growth, proliferation, metastasis, migration, invasion, apoptosis, epithelial mesenchymal transition (EMT), and doxorubicin resistance (Zhao L. et al., 2018; Zheng R. et al., 2018; Li et al., 2020e; Liu et al., 2020a; Zhang et al., 2020a).

Triple-negative breast cancer (TNBC) is a subtype of breast cancer that accounts for approximately 10–20% of total breast cancer cases (Prat et al., 2015; Bianchini et al., 2016; Medina et al., 2020). The deficiency of estrogen, progesterone, and ERBB2 receptor expression leads to its highly invasive nature and relatively low response to current therapeutics approaches. Collectively, lncRNA Xist interacts with miR-454 to inhibit cell growth in TNBC (Li et al., 2020d). And lncRNA Xist sponges with miR-92b/Slug/ESA signaling pathway to suppresses TNBC growth (Li et al., 2020c). lncRNA Xist also positively regulates PHLPP1 expression via sequestering HDAC3 from the PHLPP1 promoter to influence cells’ viability (Huang et al., 2016). In cancer immunity and brain metastasis, lncRNA Xist involves cancer immunity in high expression programmed cell death protein 1 ligand TNBC cells via activating both OCT4 and NANOG though activating PI3K/AKT/mTOR signaling pathway (Salama et al., 2019). lncRNA Xist also promotes brain metastasis in breast cancer by activating the MSN-c-Met pathway and augmenting secretion of exosomal miR-503 (Xing et al., 2018), which may serve as an effective target for the treatment of brain metastasis. These findings demonstrate LncRNA Xist may contribute to a significant approach to the treatment of breast cancer.

### LncRNA Xist in Colorectal Cancer

Colorectal cancer, with respective incidence and mortality rates of 10.2 and 9.2% in the world and which presents a rising trend in recent decades in China, ranks third in term of incidence but second in terms of mortality (Chen W. et al., 2016; Bray et al., 2018). As previously mentioned, lncRNA Xist exerts its function in colorectal cancer cells’ development by serving as a miRNA sponge. Zhang et al. (2019e) reported that lncRNA Xist, which modulates tumor size, plays a critical role in clinical prognosis and progression of colorectal cancer. Growing evidence from recent studies has shown that lncRNA Xist facilitates proliferation, metastasis, invasion, and EMT of colorectal cancer cells by functioning as an endogenous sponge of miR-200b-3p, miR-137, miR-132-3p, miR-486-5p, and miR-93-5p, thus affecting the expression of miRNAs target gene containing ZEB1, EZH2, MAPK1, NRP-2, and HIF-1A (Chen D.L. et al., 2017; Song et al., 2017; Liu et al., 2018a, 2019a; Yang L.G. et al., 2020). But beyond that, lncRNA Xist has been identified as the downstream target of methyltransferase-like14 (METTL14) by RNA-seq and Me-RIP, and its expression negatively correlating with METTL14 and YTHDF proteins 2 (YTHDF2) has been observed in colorectal cancer tissues (Yang X. et al., 2020). Yang X. et al. (2020) identified that METTL14-YTHDF2-lncRNA Xist axis mediated cells’ proliferation and metastasis in colorectal cancer.

In drug resistance of colorectal cancer cells, lncRNA Xist has been implicated in the resistance of colorectal cancer cells to chemoresistance via serving as a miRNA sponge. lncRNA Xist participates in the processes of drug resistance by modulating the axis of miR-124/serum and SGK1, miR-338-3p/PAX5, and miR-30a-5p/ROR1 (Zhu J. et al., 2018; Zhang et al., 2019d; Ma et al., 2020). Interestingly, Xiao et al. (2017) reported that overexpression of lncRNA Xist in colorectal cancer confers a potent poor therapeutic efficacy, and lncRNA Xist enjoys 5FU resistance via enhancing the expression of thymidylate synthase. In summary, this information indicates that lncRNA Xist may serve as an independent risk factor for colorectal cancer prognosis, and could be a potential therapeutic target and prognostic biomarker for colorectal cancer patients (Yu J. et al., 2020).

### LncRNA Xist in Glioblastoma

Glioblastoma (GBM), with incidence rates of 3.2 per 100,000 and relative 5-year mortality rate of just 94.9%, is the most common and lethal primary intracranial tumor with few advances in treatment over the last several decades (Batash et al., 2017; McFaline-Figueroa and Wen, 2017; Kim et al., 2018). Accumulating evidence suggests that lncRNA Xist has a pivotal role in regulating glioma cells’ properties by interacting with miRNA. lncRNA Xist affects glioblastoma development by directly binding miR-152 and miR-429 (Yao et al., 2015; Cheng Z.H. et al., 2017). However, the downstream target gene of miR-152 and miR-429 remains unclear. In addition, lncRNA Xist mediates glioma progression, tumorigenesis, metastasis, proliferation, apoptosis, and glucose metabolism by positively regulating Bcl-2, FOXC1, ZO-2, Rac1, ASCT2, SLC1A5, SOX4, Smurf1, and IRS1 by functioning as a ceRNA of miR-204-5p, miR-137, miR-133a, miR-27a, and miR-126 (Wang Z. et al., 2017; Yu H. et al., 2017; Cheng Z.H. et al., 2020; Luo C.X. et al., 2020; Shen J. et al., 2020; Sun Y. et al., 2020; Yao et al., 2020; Zhao Q. et al., 2020). In drug resistance of glioblastoma cells, lncRNA Xist has been demonstrated in the resistance of human glioblastoma cells to Temozolomide (TMZ) via the miR-29c/DNA mismatch repair (MMR) pathway (Du P. et al., 2017). And Velázquez-Flores et al. (2020) have reported that XIST and XIST-210 may act as potential biomarkers for Diffuse intrinsic pontine gliomas diagnosis and prognostic biomarkers. In summary, these findings revealed that lncRNA Xist has an oncogenic role in the tumorigenesis of glioma and may serve as a novel and potential therapeutic target for patients with glioblastoma.

### LncRNA Xist in Hepatocellular Carcinoma

Liver cancer, with respective incidence and mortality rates of 4.7 and 8.2%, was predicted to be the sixth most commonly diagnosed cancer and the fourth leading cause of cancer death worldwide in 2018 (Chen W. et al., 2016; Bray et al., 2018). The major risk factors of hepatocellular carcinoma are chronic infection with hepatitis B virus (HBV) or hepatitis C virus (HCV), aflatoxin-contaminated foodstuffs, heavy alcohol intake, obesity, smoking, and type 2 diabetes, and accounts for about 75–85% of primary live cancer (Chen W. et al., 2016; Bray et al., 2018). Recent studies have proposed that lncRNA Xist exerts tumorigenesis in hepatocellular carcinoma (Ma W.J. et al., 2017; Ma X. et al., 2017). LncRNA Xist, which functions as a ceRNA to regulate target HMGB1, SOX6, Smad7, PDK1/AKT, MAPK1, PDCD4, and PTEN expression by sponging miR-29b, miR-155-5p, miR-92b, miR-139-5p, miR-194-5p, miR-497-5p, and miR-181a, facilitates cells’ growth, autophagy, metastasis, and invasion via activating the miRNA/target signaling pathway (Zhuang et al., 2016; Chang et al., 2017; Mo et al., 2017; Kong et al., 2018; Lin et al., 2018; Xie et al., 2019; Zhang et al., 2019h). Analogously, Liu and Xu (2019) also demonstrated that silencing lncRNA Xist, whose expression level is significantly higher in hepatocellular carcinoma tissue compared with adjacent tissues, inhibits cell growth and tumor formation in hepatocellular carcinoma by directly interacting with miR-200b-3p, but the downstream target gene of miR-200b-3p remains unclear. All in all, these studies will contribute to providing a promising treatment for hepatocellular carcinoma.

### LncRNA Xist in Nasopharyngeal Carcinoma

Nasopharyngeal carcinoma, with incidence rates of 0.7% and unknown mortality rates, is relatively uncommon compared with other cancers and is one of the most common malignant tumors in the head and neck (Chua et al., 2016; Wei K.R. et al., 2017). Accumulating studies suggests that the molecular function of lncRNA Xist has a pivotal function in nasopharyngeal carcinoma properties, such as cell proliferation, migration, and invasion. Knockdown of lncRNA Xist, which negatively regulates expression of miR-29c and miR-491-5p whose target gene remains unclear, suppressed cell proliferation, invasion, and growth and induces apoptosis in nasopharyngeal carcinoma (Han et al., 2017; Cheng Q. et al., 2018). Analogously, lncRNA Xist, which is highly expressed in nasopharyngeal carcinoma tissues and cell lines, facilitates nasopharyngeal carcinoma development via activating miR-34a-5p/E2F3, miR-148a-3p/ADAM17, and miR-381-3p/NEK5 axis (Song et al., 2016; Shi et al., 2020; Zhao C.H. et al., 2020). In drug resistance of nasopharyngeal carcinoma cells, lncRNA Xist, which may present a novel and potential therapeutic target in nasopharyngeal carcinoma, has been implicated in the resistance of human nasopharyngeal carcinoma cells to cisplatin (DDP) by facilitating programmed cell death 4 (PDCD4) and Fas ligand (Fas-L) expression (Wang et al., 2019b). On the whole, these reports will be play a novel role in the treatment of nasopharyngeal carcinoma.

### LncRNA Xist in Lung Cancer

Lung cancer, with respective incidence and mortality rates of 11.6 and 18.4%, is the second most common cancer and remains the leading cause of cancer incidence and mortality worldwide (Chen W. et al., 2016; Bray et al., 2018). Emerging research demonstrates that lncRNA Xist are usually dysregulated in lung cancer and play a pivotal function in lung carcinoma initiation, progression, and therapy. lncRNA Xist, which has an oncogenic role in lung carcinoma, is closely correlated with tumor progression via regulating miR-140/iASPP axis and TCF-4 expression (Tang et al., 2017; Sun and Xu, 2019). Lung adenocarcinoma, which account for approximately 40% of total lung carcinoma, is also the most common histological subtype of NSCLC (Rong et al., 2020). lncRNA Xist expedites cancer progression and the resistance of cisplatin in lung adenocarcinoma via mediating the miR-363-3p/MDM2 and let-7i/BAG-1 signaling pathway (Sun J. et al., 2017; Rong et al., 2020). These results indicated that lncRNA Xist is likely to be a new marker and potential therapeutic target for patients with lung adenocarcinoma.

Non-small cell lung carcinoma (NSCLC), which accounts for 85% of lung cancer cases, is the most common subtype of lung cancer (Zhou et al., 2018). Accumulating evidence has revealed that lncRNA Xist is a pivotal regulator of cell proliferation, EMT, migration, invasion, and drug resistance in NSCLC. lncRNA Xist acts as an oncogene in NSCLC by modulating HIF1A-AS1 and KLF2 expression (Tantai et al., 2015; Fang et al., 2016). lncRNA Xist also positively mediates Bcl-2, LARP1, PXN and Notch-1, CBLL1, and RING1 expression by functioning as a ceRNA of miR-449a, miR-374a, miR-137, miR-212-3p, and miR-744, which are involved in cell proliferation, migration, invasion, EMT, and death in NSCLC (Xu Z.Z. et al., 2017; Zhang Y.L. et al., 2017; Jiang H.J. et al., 2018; Wang et al., 2018c, 2019c; Qiu et al., 2019). In addition, lncRNA Xist (Wang H.Y. et al., 2017), which has a higher expression in NSCLC cell lines and tissues, increases cell proliferation and invasion by negatively regulating miR-186-5p expression; however, the downstream target gene of miR-186-5p remains unclear.

It has been reported (Li C. et al., 2018) that TGF-β (Transforming growth factor β)-induced EMT serves a vital role in NSCLC metastasis and invasion. lncRNA Xist promotes TGF-β-induced EMT by positively regulating ZEB2 via interacting with miR-367 and miR-141 (Li C. et al., 2018). Analogously, lncRNA Xist inhibits NSCLC progression by sponging miR-16, miR-335, and miR-142-5p, and regulating target CDK8, SOD2/ROS, and PAX6 expression (Liu et al., 2019b; Zhou et al., 2019e; Jiang et al., 2020). Drug resistance is one of the most common reasons for therapeutic failure in patients with NSCLC and a persistent issue that requires continued investigation. Emerging evidence indicated that lncRNA Xist is associated with cisplatin resistance in NSCLC by TGF-β effector SMAD2 signaling pathway, miRNA-144-3p/MDR1 and MRP1, and miR-17/ATG7 axis (Sun W. et al., 2017; Tian et al., 2019; Xu et al., 2020). All in all, this evidence suggests that lncRNA Xist may offer a hopeful diagnostic and therapeutic choice for the treatment of NSCLC.

### LncRNA Xist in Osteosarcoma

Bone cancer, with respective incidence and mortality rates of 0.20 and 0.28%, occurs frequently in children, adolescents, and young adults aged 15 to 29 years (Siegel et al., 2019, 2020). Osteosarcoma, which accounts for 20 to 40% of all bone tumors, are the most frequent morphological subtypes of bone cancer, representing a worldwide and common primary malignant bone tumor in children and adolescents (Balmant et al., 2019; Muller and Silvan, 2019). Growing evidence from recent studies has shown that lncRNA Xist is aberrantly regulated in osteosarcoma. LncRNA Xist, which participated in osteosarcoma development processes, including cell proliferation, migration, invasion, EMT, and apoptosis, is involved in gene regulation through a variety of mechanisms, primarily by functioning as a miRNA sponge and via interacting with its targets (Li et al., 2017; Wang et al., 2019f; Han and Shen, 2020), such as miR-153/SNAI1 pathway (Wen et al., 2020), EZH2, PUMA, and NF-kB (Xu T. et al., 2017; Gao et al., 2019).

In addition to indirect modulation of ceRNAs, studies published to date have indicated that high lncRNA Xist expression in osteosarcoma results in sponging six miRNAs, namely miR-21-5p, miR-193a-3p, miR-195-5p, miR-320b, miR-137, and miR-375-3p, which affects osteosarcoma progression (Wu D.P. et al., 2017; Zhang and Xia, 2017; Lv et al., 2018; Yang C. et al., 2018; Li H. et al., 2019; Sun X. et al., 2019). lncRNA, which regulates miR-21-5p/PDCD4 axis, miR-193a-3p/RSF1 axis, miR-195-5p/YAP axis, miR-137, miR-320b/RAP2B axis, and miR-375-3p/KT/mTOR axis, contributes to osteosarcoma cell growth, metastasis, and invasion by activating MAPK signaling pathway, NF-kB signaling pathway, and PI3K-AKT-mTOR signaling pathway. Taking all into account, these studies indicated that lncRNA Xist may act as a candidate prognostic biomarker and a promising therapeutic target for osteosarcoma (Wu D.P. et al., 2017; Zhang and Xia, 2017; Lv et al., 2018; Yang C. et al., 2018; Li H. et al., 2019; Sun X. et al., 2019).

### LncRNA Xist in Pancreatic Cancer

Pancreatic cancer, with respective incidence and mortality rates of 2.5% (China, 2.1%, 2015) and 4.5% (China, 2.8%, 2015), was the seventh leading cause of cancer death worldwide in both males and females in 2018 (Chen W. et al., 2016; Bray et al., 2018). Accumulating evidence indicated that lncRNA Xist interacts with additional miRNAs, such as miR-133a, miR-140 and miR-124, miR-34a-5p, miR-34a, miR-141-3p, and miR-429 in pancreatic cancer, and is involved in the development and progression of pancreatic cancer (Liang et al., 2017; Wei W. et al., 2017; Sun Z. et al., 2018; Shen et al., 2019; Sun and Zhang, 2019; Zou et al., 2020). As aforementioned, lncRNA Xist promotes pancreatic cancer cells’ proliferation by binding miR-133a, thus affecting the miR-133a downstream target gene EGFR (epidermal growth factor receptor) which is positively correlated with lncRNA Xist (Wei W. et al., 2017). lncRNA Xist also facilitates miR-140/miR-124/iASPP/CDK1 axis, miR-34a/YAP axis, miR-141-3p/TGF-β2 axis, miR-429/ZEB1 axis and miR-34a-5p, which contributes to carcinoma cell growth, EMT, migration, and invasion (Liang et al., 2017; Sun Z. et al., 2018; Shen et al., 2019; Sun and Zhang, 2019; Zou et al., 2020). However, the downstream target gene of miR-34a-5p remains unknown. Taken together, the above research results suggested that lncRNA Xist could be regarded as a candidate prognostic biomarker and a potential therapeutic target in human pancreatic carcinoma.

### LncRNA Xist in Retinoblastoma

Retinoblastoma, which has a significant effect on mortality in emerging countries but is more curable in industrialized countries, is an aggressive eye cancer that affects infants and children (Cassoux et al., 2017). Recently, abundant studies demonstrated that dysregulation lncRNA was involved in tumorigenesis and cancer progression of retinoblastoma (Yang and Wei, 2019). Compared to healthy controls, lncRNA Xist was significantly upregulated in plasma of retinoblastoma patients which was inversely associated with lncRNA NKILA (Lyu et al., 2019). LncRNA Xist overexpression promotes retinoblastoma cells proliferation, migration, and invasion rates via negatively regulating lncRNA NKILA, but the causality has not been fully validated. In addition, lncRNA Xist, which indirectly interacts with miR-21-5p, miR-124, miR-101, miR-140-5p, and miR-200a-3p, and positively regulates VEGF, STAT3, ZEB1 and ZEB2, SOX4, and NRP1 expression, facilitates apoptosis, migration, EMT, proliferation, and invasion by activating signaling pathways, such as PI3K-Akt signaling pathway and MAPK-ERK signaling pathway (Hu et al., 2018; Cheng Y. et al., 2019; Dong et al., 2020; Wang et al., 2020c; Zhao H. et al., 2020). All in all, these studies suggested that lncRNA Xist serves a potential and promising clinical application for diagnosis, prognosis, and treatment.

### LncRNA Xist in Cervical Cancer

Cervical cancer, with respective incidence and mortality rates of 3.2 and 3.3%, ranked as the fourth most frequently diagnosed cancer and the fourth leading cause of cancer death in women in 2018 worldwide (Chen W. et al., 2016; Bray et al., 2018). Recently, numerous reports found that dysregulation of lncRNA Xist was involved in regulating cervical cancer progression via binding to miRNAs. Zhu H. et al. (2018) demonstrated lncRNA Xist, which is extremely highly expressed in cervical cancer tissues and cell lines, accelerates cervical cancer progression via upregulating Fus through functioning as a ceRNA of miR-200a. In additional, lncRNA Xist upregulation, which positively facilitates ORC1 expression and acts as a ceRNA of miR-140-5p, contributes to the cervical cancer progression by activating miR-140-5p/ORC1 axis (Chen X. et al., 2019c). Similarly, Liu et al. (2020d) found that lncRNA Xist, which was highly expressed in cervical cancer cells and tissue, promoted cervical cancer cells’ proliferation, migration, and invasion and hindered apoptosis by inhibiting miR-889-3p and positively mediating SIXI expression. Taken together these studies demonstrated that lncRNA Xist may play a role in epigenetic diagnostics and therapeutics in cervical cancer.

### LncRNA Xist in Gastric Cancer

Stomach cancer (cardia and non-cardiac gastric cancer combined), with respective incidence and mortality rates of 5.7 and 8.2%, was the fifth most frequently diagnosed cancer and the third leading cause of cancer death in 2018 worldwide, and remains an important cancer (Chen W. et al., 2016; Bray et al., 2018). Recently, some reports founded that lncRNA Xist exerts its function in gastric cancer progression by acting as a miRNA sponge, It acts on miRNA, such as miR-101, miR-497, miR-185, and miR-337. lncRNA Xist, which acts as a molecular sponge of miR-101, miR-497, miR-185, and miR-337 to mediate EZH2, MACC1, TGF-β1, and JAK2 expression, is involved in gastric cancer progression through mediating miR-101/EZH2 axis, miR-497/MACC1 axis, miR-185/TGF-β1 axis, and miR-337/JAK2 axis (Chen D.L. et al., 2016; Ma L. et al., 2017; Zhang Q. et al., 2018; Zheng W. et al., 2020). In drug resistance of gastric carcinoma cells, Li Y.D. et al. (2019) demonstrated that lncRNA Xist contributes to drug resistance of gastric cancer cells though positively facilitating the related gene MDR1 (multidrug resistance gene 1) and MRP1 (multi-drug resistance protein 1) of multidrug resistance, and is helpful for the molecule-targeted treatment of gastric cancer. Taken together, these findings suggest that lncRNA Xist may be a candidate prognostic biomarker and a new therapy target in gastric cancer patients.

### LncRNA Xist in Melanoma

Melanoma, with respective incidence and mortality rates of 1.6 and 0.6%, was the most fatal form of skin cancer in 2018 worldwide and the rates are increasing faster than any other currently preventable cancers (Chen W. et al., 2016; Bray et al., 2018; Schadendorf et al., 2018). Recently, some findings suggested that a major role of lncRNA Xist is facilitating melanoma progression via acting as a miRNAs sponge to regulate its downstream target genes, such as lncRNA Xist (Zhang et al., 2019f), which promoted malignant melanoma growth and metastasis by functioning as a ceRNA though miR-217. However, the downstream target gene of miR-217 remains unclear. Analogously, lncRNA Xist (Pan et al., 2019; Tian K. et al., 2020), which functions as a ceRNA to positively regulate ROCK1 and PI3KRI and AKT expression by sponging miR-139-5p and miR-21, respectively, facilitates proliferation, invasion, and oxaliplatin resistance of melanoma cells. In summary, this evidence shows that lncRNA Xist could provide a novel insight into the pathogenesis and underlying therapeutic targets for melanoma.

### LncRNA Xist in Esophageal Cancer

Esophageal cancer, with respective incidence and mortality rates of 3.2 and 5.3%, ranks seventh in terms of incidence and sixth in mortality overall, the latter signifying that esophageal cancer was responsible for an estimated 1 in every 20 cancer deaths in 2018 worldwide (Chen W. et al., 2016; Bray et al., 2018; Schadendorf et al., 2018). Recently, numerous reports demonstrated that dysregulation of lncRNA Xist was involved in regulating esophageal cancer development via binding to miRNAs. lncRNA Xist involves esophageal squamous cell carcinoma development via regulation of miR-101/EZH2 axis (Wu X. et al., 2017), and facilitates esophageal squamous cell carcinoma proliferation, apoptosis, migration, and invasion via regulation miR-494/CDK6 axis and activation of JAK2/STAT3 signal pathway (Chen et al., 2019f). In addition, lncRNA Xist predicts the presence of lymph node metastases in human esophageal squamous cells (Li and He, 2019; Wang et al., 2020b). Taken together, these results demonstrated that lncRNA Xist may provide a novel candidate prognostic biomarker and a new insight for esophageal carcinoma therapy.

### LncRNA Xist in Laryngeal Squamous Cell Carcinoma

Laryngeal cancer, with respective incidence and mortality rates of 1.0 and 1.0%, was one of the most common tumors of the respiratory tract in 2018 worldwide (Chen W. et al., 2016; Steuer et al., 2017; Bray et al., 2018). Squamous cell carcinoma, accounting for approximately 90% of malignant neoplasms of the larynx, is the most common malignancy of the larynx (Thompson, 2017; Bradford et al., 2020). Recently, some evidence suggested that a major role of lncRNA Xist, which is notably up-regulated in laryngeal squamous cell carcinoma tissues and cells, promotes laryngeal squamous cell carcinoma progression via interacting with miRNAs to regulate its downstream target gene. lncRNA Xist increases the aggressiveness of laryngeal squamous cell carcinoma by functioning as a ceRNA sponge of miR-124 to regulate EZH2 expression (Xiao D. et al., 2019), and promotes progression of laryngeal squamous cell carcinoma via activating the miR-144/IRS1 axis (Cui et al., 2020), and promotes the malignance of laryngeal squamous cell carcinoma cells through functioning as a ceRNA of miR-125b-5p to positively modulate TRIB2 expression (Liu et al., 2020b). All together, these studies demonstrated that lncRNA Xist may serve as a new potential prognostic biomarker and putative target in the therapy of laryngeal squamous cell carcinoma.

### LncRNA Xist in Ovarian Cancer

Ovarian cancer, which is the leading cause of death for women of reproductive age around the world and has a 5-year survival rate below 45%, is in eighth place among the most common cancers in women and the fifth leading cause of death among women worldwide, including 4% of all cancers (Webb and Jordan, 2017; Moga et al., 2018; Stewart et al., 2019). In order to prove the lncRNA Xist participated in ovarian cancer development, Wang et al. (2018a) revelated that lncRNA Xist is involved in ovarian cancer development by negatively regulating miR-214-3p expression. This confirmed that lncRNA Xist is closely associated with the tumor grade and distant metastasis in the ovarian cancer patients (Zuo et al., 2019). This result suggested that lncRNA Xist plays a role in tumor development. In addition, lncRNA Xist (Wang and Li, 2020), which functions as a ceRNA to positively mediate the expression of PDCD4 (programmed cell death protein 4) through binding to miR-150-5p and is significantly decreased in ovarian cancer tissues and cell lines compared with the normal tissue and cells, inhibits ovarian cancer cell growth and metastasis via regulating miR-150-5p/PDCD4 signaling pathway. All in all, these studies evaluated that lncRNA Xist provides insight into the potential target for the treatment of ovarian cancer, and a new evaluation of the diagnosis and prognosis of ovarian cancer.

### LncRNA Xist in Others Cancer

Growing evidence from recent studies has shown that lncRNA Xist facilitates tumor development, including pituitary neuroendocrine tumor, neuroblastoma, thyroid cancer, colon cancer, renal cell carcinoma, and prostate cancer (Chaligne and Heard, 2014; Yang Z. et al., 2018; Liu et al., 2019c). In the pituitary neuroendocrine tumor cells (Zhou et al., 2019b), lncRNA Xist, which functions as a ceRNA to sequester miR-424-5p to elevate the expression of the its target bFGF, and exhibits high expression in invasive pituitary neuroendocrine tumor tissues as compared to non-invasive tumor tissues, promotes cancer progression in invasive pituitary neuroendocrine tumor via activating the miR-424-5p/bFGF signaling pathway. In neuroblastoma (Zhang et al., 2019a), lncRNA Xist, which interacts with EZH2 to downregulate DKK1 by inducing H3 histone methylation, promotes neuroblastoma cell growth, proliferation, migration, and invasion via modulating H3 histone methylation of DKK1 in neuroblastoma. In addition, lncRNA Xist (Yang H. et al., 2020) repressed tumor growth and boosted radiosensitivity of neuroblastoma via modulating the miR-375/L1CAM axis. In thyroid cancer, lncRNA Xist (Liu et al., 2018c), which positively regulates MET by sponging miR-34a, modulates the cell proliferation and tumor growth through activating the PI3K/AKT signaling pathway. Analogously, Xu Y. et al. (2018) demonstrated that lncRNA Xist, whose high expression is positively associated with TNM stage and lymph node metastasis, promotes cell proliferation and invasion by interacting with miR-141 in papillary thyroid carcinoma. However, the downstream target gene of miR-141 remains unknown. In colon cancer cells, lncRNA Xist (Sun N.N. et al., 2018), which functions as a ceRNA by binding to miR-34a and positively modulates WNT1 expression, has a crucial function in colon cancer progression via the miR-34a/WNT1 axis to activate the Wnt/β-catenin signaling pathway.

In addition to indirect regulation of ceRNAs, studies published to date have manifested that low lncRNA Xist expression in renal cell carcinoma results in sponging two miRNAs, miR-106b-5p and miR-302c, which regulates tumor development (Zhang J. et al., 2017; Sun K. et al., 2019). lncRNA Xist, which positively facilitates P21 and SDC1 expression through sponging miR-106b-5p and miR-302c, facilitates cell proliferation and apoptosis via miR-106b-5p/P21 signaling pathway and miR-302c/SDC1 axis. In prostate cancer cells, lncRNA Xist, which weakly expresses in normal prostate tissues but not in leukocytes, contributes prostate cancer development (cell proliferation and metastasis) by activating miR-23a/RKIP signaling pathway (Laner et al., 2005; Du Y. et al., 2017). In addition, SQ. Hai (2020) have indicated that LncRNA XIST/miR-124-3p/iASPP Pathway Promotes Growth of Human Chordoma Cells. Analogously, Lobo et al. (2019) manifested that demethylated and methylated XIST promoter may be involved in testicular germ cell tumor development. Altogether, this evidence pronounced that lncRNA Xist may shed new light on epigenetic diagnostics and therapeutics for cancer patients.

## The Role of LncRNA Xist in Non-Cancer Diseases

Diseases are abnormal conditions that have a specific set of signs and symptoms. Diseases can have an external cause, such as an infection, or an internal cause, such as autoimmune disease (such as Alzheimer’s disease). Accumulating evidence has suggested that lncRNA Xist participates in non-cancer related diseases’ development and progression (Table 2) as a ceRNA regulatory network of miRNA-mRNA. It also provides a window into the understanding of aberrant expression of lncRNA Xist associated with non-cancer related diseases. lncRNA Xist is a novel potential biomarker and could potentially be involved in the diagnosis and therapy of different types of diseases.

**TABLE 2:** LncRNA Xist and miRNA in non-cancer related disease.

**Disease type**	**miRNA**	**Target**	**Mechanism of action and function**	**Signaling pathway**	**References**
Cardiac disease	miR-330-3p, miR-101	S100B, TLR2	Aberrant expression lncRNA Xist is involved in non-cancer related diseases and cells development, such as cell apoptosis, cell cycle, cell proliferation, cell differentiation.	TGF-beta signaling pathway, PI3K-Akt signaling pathway, Toll-like receptor signaling pathway, cAMP signaling pathway, Notch signaling pathway, Prolactin signaling pathway, JAK-STAT signaling pathway, Toll-like receptor signaling pathway, NF−κB signaling pathway, NOD-like receptor signaling pathway, C-type lectin receptor signaling pathway, Hedgehog signaling pathway, Thyroid hormone signaling pathway, HIF-1 signaling pathway, Wnt/β-catenin signaling pathway, BMP/TGF-β signaling pathway, MAPK and MMPs signaling pathway, Human papillomavirus infection, AGE-RAGE signaling pathway in diabetic complications, Relaxin signaling pathway, T cell receptor signaling pathway and B cell receptor signaling pathway, etc.	Chen Y. et al., 2018; Xiao L. et al., 2019
Myocardial infarction	miR-130a-3p, miR-101a-3p	PDE4D, FOS			Zhou et al., 2019d; Lin B. et al., 2020
Acute myocardial infarction	miR-449, miR-122-5p, miR-125b, miR-133a, miR-150-5p	Notch1, FOXP2, hexokianse 2, SOCS2, Bax			Li Z.Q. et al., 2019; Zhang et al., 2019c; Fan et al., 2020; Peng et al., 2020; Zhou et al., 2020
Neuropathic pain	miR-133b-3p, miR-154-5p, miR-137, miR-544, miR-150	Trisomy 21, Pitx3, TLR5, TNFAIP1, STAT3, ZEB1			Jin et al., 2018; Yan et al., 2018; Zhao Y. et al., 2018; Wei et al., 2019
Neurodegeneration	miR-133b-3p	Pitx3			He et al., 2020
Alzheimer’s disease	miR-132, miR-124	BACE1			Wang et al., 2018b; Du Y. et al., 2020
Osteoarthritis	miR-211, miR-214-3p, miR-17-5p, miR-1277-5p, miR-376c-5p, miR-142-5p, miR-149-5p, miR-675-5p	CXCR4, MAPK, AHNAK, BMP2, TIMP-3, MMP-13, ADAMTS5, OPN, SGTB, DNMT3A, GNG5			Li L. et al., 2018; Liao et al., 2019; Wang et al., 2019e; Feng et al., 2020; Ghaderian et al., 2020; Li et al., 2020b; Liu et al., 2020e; Shen X.F. et al., 2020
Bone marrow	miR-9-5p	ALPL, ALP, Bglap, Runx2			Zheng C. et al., 2020
Inflammation	miR-27a-3p, miR-34a, miR-30c-5p, miR-146a	NF−κB, NLRP3, Smurf1, YY1, PTEN, Nav1.7			Shenoda et al., 2018; Sun W.B. et al., 2018; Hu W.N. et al., 2019; Zhao Q. et al., 2020
Spinal cord injury	miR-27a, miR-494, miR-32-5p	Smurf1, PTEN, Notch-1			Gu et al., 2017; Cheng X. et al., 2020; Zhao Q. et al., 2020
Acute kidney injury	miR-15-5p, miR-212-3p, miR-122-5p, miR-142-5p	CUL3, ASF1A, BRWD1M, PFKFB2, PDCD4			Xu et al., 2019; Cheng and Wang, 2020; Tang et al., 2020
Nephropathy	miR-217, miR-93-5p, miR-485	TLR4, CDKN1A, PSMB8			Jin et al., 2019; Yang et al., 2019; Wang, 2020
Placental angiogenesis	miR-429, miR-485-3p	VEGF-A, SOX7, ERK1/2, Akt			Chen G. et al., 2018; Hu C. et al., 2019
Acute pneumonia	miR-370-3p	TLR4, JAK, STAT, NF−κB			Zhang et al., 2019g
Pulmonary fibrosis	miR-139	β-catenin			Wang Y.C. et al., 2017
Primary graft dysfunction	miR-21	IL-12A			Li et al., 2020a
Rett syndrome		MeCP2, BMP/TGF-β			Sripathy et al., 2017; Adrianse et al., 2018
Acute respiratory distress syndrome	miR-204	IRF2			Wang et al., 2019d
SCNT embryo development		REX1, YY1, MSL1/MSL2			
Human trophoblast cells	miR-144	Titin, MAPK, MMPs			Yu N.H. et al., 2017
Endothelial cells injury	miR-320	NOD2			Xu X.H. et al., 2018
Skin fibroblasts	miR-29a, miR-29b-3p	LIN28A, COL1A1			Guo et al., 2018; Cao and Feng, 2019
Osteoblasts	miR-203-3p, let-7c-5p	ZFPM2, STAT3			Niu et al., 2020; Wang et al., 2020d
Keratoconus	miR-181a	COL4A1			Tian R. et al., 2020
Hair follicle regeneration	miR-424	Shh			Lin B.J. et al., 2020
Acute liver injury		BRD4			Shen and Li, 2020
Stanford Type A Aortic Dissection	miR-17	PTEN			Zhang et al., 2020b

### LncRNA Xist in Cardiac Disease

Cardiac diseases, including coronary artery disease (CAD), myocardial infarction (MI), cardiac hypertrophy, and heart failure (HF), are among the leading causes of morbidity and mortality worldwide (Greco et al., 2018; Colpaert and Calore, 2019). Emerging evidence has revealed that lncRNA Xist acted as powerful and dynamic modifier of cardiac physiological and pathological processes. Cardiac hypertrophy, recognized as a risk predictor of sudden cardiac death, is an adaptive reaction in response to altered stress or injury to maintain cardiac function (Li Y. et al., 2018; Wehbe et al., 2019; Luo X. et al., 2020). lncRNA Xist (Sohrabifar, 2020) participates in the pathogenesis of complex diseases and also serves as a diagnostic marker. lncRNA Xist also positively regulates S100B expression through functioning as a ceRNA to bind miR-330-3p (Chen Y. et al., 2018) and functions as a ceRNA of miR-101 to enhance TLR2 expression (Xiao L. et al., 2019) and modulates the progression of cardiomyocyte hypertrophy by miR-330-3p/S100B pathway and miR-101/TLR2 axis.

Myocardial infarction (MI), colloquially known as “heart attack,” is caused by decreased or complete cessation of blood flow to a portion of the myocardium and by the rupture of atherosclerotic plaques, which results in damage to cardiomyocytes due to lack of oxygen (Colpaert and Calore, 2019; Ojha and Dhamoon, 2020). lncRNA Xist, which positively mediates PDE4D expression via interacting miR-130a-3p (Zhou et al., 2019d) and targets miR-101a-3p through regulating FOS (Lin B. et al., 2020), promotes myocardial infarction development and cell apoptosis, and inhibits cell proliferation though the miR-130a-3p/PDE4D aixs and miR-101a-3p/FOS aixs.

Acute MI (AMI) is characterized by ischemic injury and cardiomyocyte apoptosis, while myocardial injury, which is also an entity in itself, is a prerequisite for the diagnosis of MI in the setting of acute myocardial ischemia (Sandoval and Thygesen, 2017; Sandoval et al., 2017; Colpaert and Calore, 2019). lncRNA Xist, which interacts directly with miRNA (miR-150-5p, miR-122-5p, miR-125b, miR-133a, and miR-449) to positively regulate expression levels of mRNA (Bax, FOXP2, hexokianse 2, SOCS2, and Notch1), protects hypoxia-induced cardiomyocyte injury and represses the myocardial cell apoptosis though miR-150-5p/Bax pathway, miR-122-5p/FOXP2 axis, miR-125b/hexokianse 2 axis, miR-133a/SOCS2 pathway, and miR-449/Notch1 signaling pathway (Li Z.Q. et al., 2019; Zhang et al., 2019c; Fan et al., 2020; Peng et al., 2020; Zhou et al., 2020). These results indicated that lncRNA Xist represents a very promising potential pharmacotherapeutic target and biomarker for cardiac disease.

### LncRNA Xist in Neuropathic Pain

Neuropathic pain, including central pain, peripheral pain, and cancer pain, is pain that arises as lesions or diseases of the somatosensory system, either at the peripheral or at the central level, and are treated by first-line (include antidepressants and anticonvulsants acting at calcium channels), second-, and third-line (include topical lidocaine and opioids) pharmacologicals (Xu et al., 2016; Fornasari, 2017; Eberlin, 2019). Growing studies have revealed that lncRNA Xist, which has been characterized as a key modulator of neuronal functions, plays a pivotal role in the development of neuropathic pain. In Down’s syndrome, lncRNA Xist (Czerminski and Lawrence, 2020), which fully corrects trisomy 21 dosage in neural cells, promotes differentiation of trisomic NSCs (neural stem cells) to neurons by silencing Trisomy 21 and activating Notch signaling pathway. In Parkinson’s disease (PD) animals, it has been shown that lncRNA Xist/miR-133b-3p/Pitx3 axis protect dopaminergic neurons through activation of CB2R with AM1241, which alleviates PD (He et al., 2020). In addition, lncRNA Xist participated in neuropathic pain though interacting with miRNAs in CCI (chronic constriction injury) rat models, including miR-154-5p, miR-137, miR-544, and miR-150. lncRNA Xist, which functions as a ceRNA to positively modulate mRNA expression (TLR5, TNFAIP1, STAT3, and ZEB1) by sponging miRNA (miR-154-5p, miR-137, miR-544, and miR-150), contributes to neuropathic pain development by facilitating miR-154-5p/TLR5 axis, miR-137/TNFAIP1 axis, miR-544/STAT3 axis, and miR-150/ZEB1 axis in CCI rat models (Jin et al., 2018; Yan et al., 2018; Zhao Y. et al., 2018; Wei et al., 2019).

Alzheimer’s disease (AD), which is a growing global health concern with huge implications for individuals and society, is a chronic progressive and irreversible neurodegenerative disorder (Scheltens et al., 2016; Lane et al., 2018; Chanda and Mukhopadhyay, 2020). Silencing lncRNA Xist (Wang et al., 2018b) attenuated Aβ(amyloid-beta peptide)25-35-induced toxicity, oxidative stress, and apoptosis in primary cultured rat hippocampal neurons by negatively mediating miR-132 expression. But the downstream target gene of miR-132 remains unclear. Similarly, Du Y. et al. (2020) showed that lncRNA Xist, which was significantly upregulated in hydrogen peroxide (H_2_O_2_)-induced AD mice models and in H_2_O_2_-treated N2a cells, is involved in Alzheimer’s disease development though positively regulating BACE1 expression by interacting with miR-124. These studies suggested that lncRNA Xist might provide novel therapeutic avenues for neuropathic diseases.

### LncRNA Xist in Osteoarthritis

Osteoarthritis (OA), which is the most common joint disorder that affects one or several diarthrodial joints including small joints (such as those in the hand) and large joints (such as the knee and hip joints), is the most frequently diagnosed musculoskeletal disease and leads to functional decline and loss in quality of life (Kraus, 2014; Pereira et al., 2015). Accumulated evidence manifested that lncRNA Xist is associated with development and progression of OA. lncRNA Xist, which acts as a ceRNA of miR-211 to positively mediate miR-211-interacted CXCR4 expression, promotes the proliferation and apoptosis of OA through the miR-211/CXCR4 axis activating MAPK signaling pathway (Li L. et al., 2018). And lncRNA Xist (Liao et al., 2019), which positively regulates AHNAK expression to activate BMP2 Signaling Pathway by target with miR-17-5p, may influence Cervical Ossification of the PLL through facilitating of miR-17-5P/AHNAK/BMP2 axis. In periodontal ligament stem cells (PDLSCs), lncRNA Xist, which was elevated in osteogenic inducted PDLSCs, promoted Osteogenic Differentiation by negatively regulating the expression of miR-214-3p, but the downstream target gene of miR-214-3p remains unknown (Feng et al., 2020).

In osteoporosis (OP), lncRNA Xist (Chen et al., 2019a,d), which was highly expressed in the serum and monocytes of patients with OP, regulates osteoporosis through recruiting DNA methyltransferase and inhibiting bone marrow mesenchymal stem cell differentiation. In addition, a major role of lncRNA Xist is facilitating gene expression and affecting osteoarthritis development and progression via sponging to miRNAs. The lncRNA Xist/miR-9-5p/ALPL (Zheng C. et al., 2020) and lncRNA Xist/miR-1277-5p/MMP-13 and ADAMTS5 (Wang et al., 2019e) signaling pathway has been identified as a ceRNA regulatory network involved in osteoarthritis development. And other ceRNA regulatory networks have also been shown to contribute to the progression of Osteoarthritis, such as lncRNA Xist/miR-376c-5p/OPN signaling pathway (Li et al., 2020b), lncRNA Xist/miR-142-5p/SGTB signaling pathway (Ghaderian et al., 2020), lncRNA Xist/miR-149-5p/DNMT3A signaling pathway (Liu et al., 2020e), and lncRNA Xist/miR-675-3p/GNG5 signaling pathway (Shen X.F. et al., 2020). Increasing studies have shown that lncRNA Xist might act as a novel therapeutic target for OA patients.

### LncRNA Xist in Inflammation

Inflammation, which is activated by inflammasomes that are innate immune system receptors and sensors that regulate the activation of caspase-1, is a protective immune response mounted by the evolutionarily conserved innate immune system in response to harmful stimuli, such as pathogens, dead cells, or irritants, and is tightly regulated by the host (Guo et al., 2015). Recent findings demonstrated the pivotal role of lncRNA Xist in the progression of the inflammatory response. NF-κB (nuclear factor-κB) signaling pathway, which plays a vital role in inflammation and innate immunity, were involved in cell proliferation and apoptosis and regulated the production of inflammatory cytokines including tumor necrosis factor (TNF)-α, interleukin (IL)-1β, IL−6, and IL−8 (Ma et al., 2019; Shenoda et al., 2020). lncRNA Xist facilitates acute inflammatory responses and bovine mammary epithelial cell inflammatory responses via NF-κB/NLRP3 inflammasome signaling pathway (Levey and James, 2017; Yang et al., 2019).

In addition to the indirect regulation of ceRNAs, studies published to date have demonstrated that high lncRNA Xist expression in inflammatory cells results in sponging four miRNAs, namely miR-27a-3p, miR-30c, miR-34c, and 146a, which responded to the inflammatory development process (Shenoda et al., 2018; Sun W.B. et al., 2018; Hu W.N. et al., 2019; Zhao Q. et al., 2020). The regulation of lncRNA Xist in most inflammation processes, including acute inflammation response in female cells, apoptosis, and inflammatory injury of microglia cells after spinal cord injury, cell apoptosis of HUVEC (Human umbilical vein endothelial cells) and ox-LDL (oxidized low-density lipoprotein)-induced the inflammatory response and inflammatory pain in rat. The pathways involved included lncRNA Xist/miR-34a/YY1 signaling pathway (Shenoda et al., 2018), lncRNA Xist/miR-27a/Smurf1 signaling pathway (Zhao Q. et al., 2020), lncRNA Xist/miR-30c-5p/PTEN signaling pathway (Hu W.N. et al., 2019), and miR-146a/Nav1.7 signaling pathway (Sun W.B. et al., 2018). These results suggest that lncRNA Xist could be involved in a promising strategy against inflammation and be a potential target for inflammatory patients.

### LncRNA Xist in Kidney and Cardiovascular Disease

As the kidney and heart are intricately linked, abnormal function in one can lead to pathological function in the other (Lorenzen and Thum, 2016). Acute kidney injury (formerly known as acute renal failure), which is typically diagnosed by the accumulation of end products of nitrogen metabolism (urea and creatinine) or decreased urine output, or both, is a syndrome characterized by the rapid loss of the function of glomerular filtration rate (Levey and James, 2017; Ronco et al., 2019). Recent studies indicated lncRNA Xist exerts its function by serving as a miRNA sponge in the development of kidney injury. In diabetic nephropathy, lncRNA Xist, which is highly expressed in the kidney tissue of diabetic nephropathy mice and high glucose-exposed HK-2 cells, is involved in diabetic nephropathy development by positively facilitating CDKN1A (cyclin-dependent kinase inhibitor 1A) expression via functioning as a ceRNA of miR-93-5p (Yang et al., 2019). In LPS-induced SCI (Spinal cord injury) microglia cells and lncRNA Xist, which interacts with miR-27a to mediate the downstream target gene of Smurf1 expression, alleviated the apoptosis and inflammatory injury of microglia cells after SCI through activating miR-27a/Smurf1 axis (Zhao Q. et al., 2020). These signaling pathways, which include lncRNA Xist/miR-494/PTEN/PI3K/AKT signaling pathway (Gu et al., 2017), lncRNA Xist/miR-142-5p/PDCD4 signaling pathway (Tang et al., 2020), lncRNA Xist/miR-217/TLR4 signaling pathway (Jin et al., 2019), lncRNA Xist/miR-32-5p/Notch-1 signaling pathway (Cheng X. et al., 2020), and lncRNA Xist/miR-15a-5p/CUL3signaling pathway (Xu et al., 2019), participated in the SCI, acute kidney injury, and nephropathy procession. Cheng and Wang (2020) identified that lncRNA Xist could act as a ceRNA to sponge miR-212-3p and miR-122-5p to facilitate kidney transplant acute kidney injury progression via regulating the expression of ASF1A, BRWD1, and PFKFB2 using GEO database assay.

In contrast to kidney diseases, the study of lncRNA Xist in cardiovascular diseases is still in its infancy. Chen G. et al. (2018) suggested that lncRNA Xist, which is disrupted by aberrant expression of PFOS (Perfluorooctane sulfonate) in prenatal cells, facilitates placental angiogenesis by regulation of miR-429/VEGF-A axis. Similarly, lncRNA Xist (Hu C. et al., 2019), which positively modulates SOX7 (SRY-box 7) expression by sponging miR-485, participated in hypoxia-induced angiogenesis to activate VEGF signaling pathway, ERK1/2, and Akt signaling pathway through regulation miR-485/SCX7 axis. In additional, Stanford Type A Aortic Dissection (TAAD) is one of the most lethal cardiovascular diseases with an extremely high morbidity and mortality rate. Zhang et al. (2020b) have suggested that lncRNA Xist, which positively regulates PTEN expression via its competitive target miR-17, modulates the proliferation and apoptosis of vascular smooth muscle cells to affect Stanford Type A Aortic Dissection. All in all, these findings provide a new orientation for lncRNA Xist in kidney and cardiovascular diseases.

### LncRNA Xist in Other Disease and Cells

A growing number of studies exhibited (Agrelo and Wutz, 2010; Shi et al., 2013; Cantone and Fisher, 2017) that lncRNA Xist participated in disease-associated processes, such as pulmonary disease, diabetic nephropathy, dermal diseases, and hereditary diseases, and mediated cellular functions of cells, such as somatic cell, B cells, and embryonic stem (ES) cells. In acute pneumonia, lncRNA Xist was robustly increased in serum of patients with acute-stage pneumonia and LPS (lipopolysaccharide)-induced WI-38 (normal human fibroblast WI-38 cell line) human lung fibroblasts cells, which shows it is involved in the progression of cell inflammatory response (Zhang et al., 2019g). Consequently, knockdown lncRNA Xist, which functions as a ceRNA to positively modulate TLR4 expression by sponging miR-370-3p, remarkably alleviates LPS-induced cell injury through regulating miR-370-3p/TLR4 axis to activate JAK/STAT and NF−κB signaling pathways (Zhang et al., 2019g).

In pulmonary fibrosis, Wang Y.C. et al. (2017) have revealed that lncRNA Xist regulates bleomycin (BLM)-induced extracellular matrix (ECM) and pulmonary fibrosis via modulation of miR-139/β-catenin axis. Primary graft dysfunction (PGD), which is a major cause of fatality post-lung transplantation, is a known acute lung injury (ALI). Li et al. (2020a) found that lncRNA Xist, which positively elevates the expression of IL-12A by acting as a ceRNA of miR-21, induces NET (neutrophil extracellular trap) formation and accelerates PGD after lung transplantation by activating the network of miR-21/IL-12A.

Diarrhea-predominant irritable bowel syndrome (IBS-D) is prevalent and has a high incidence rate in children. Zhang et al. (2020d) have demonstrated that lncRNA Xist, which is highly expressed in visceral hypersensitivity mice with IBS-D, modulates HT (5-hydroxytrytophan)-induced visceral hypersensitivity by epigenetic silencing of the SERT gene in mice with diarrhea-predominant IBS. In addition, Shen X.F. et al. (2020) have suggested that the silencing of lncRNA Xist, which is highly expressed in serum of patients, protects against sepsis-induced acute liver injury via inhibition of BRD4 expression. In diabetic nephropathy, Wang (2020) reported that lncRNA Xist silencing, which positively modulates PSMB8 expression via acting as a sponge for miR-485 in HMCs (human mesangial cells) treated with high glucose, alleviates inflammation and mesangial cell proliferation via interacting with miR-485/PSMB8.

Autoimmune disorders, such as Hashimoto’s thyroiditis, Sjögren’s Syndrome, systemic lupus erythematosus (SLE), and Grave’s disease, where 85–95% of patients are women, exhibited a strong female bias (Syrett et al., 2019, 2020). In recent years, lncRNA Xist (Syrett et al., 2017, 2019, 2020; Zhang et al., 2020c), which serves a vital function in SLE by RNA-seq data, promotes SLE development in NZB/WF1 mice with lupus-like disease. Taken together, these studies provide an important insight into how lncRNA Xist provides a therapeutic opportunity in female-biased autoimmune disorders.

Rett syndrome (RS), which is a debilitating neurological disorder affecting mostly girls, was caused by heterozygous mutations in the gene encoding the methyl-CpG–binding protein MeCP2 on the X chromosome (Sripathy et al., 2017). lncRNA Xist facilitates RS development through regulation of the bone morphogenetic protein (BMP)/TGF-β signaling pathway (Sripathy et al., 2017), and contributes to mouse brain development through reactivating MeCP2 expression (Adrianse et al., 2018).

Acute respiratory distress syndrome (ARDS), which is associated with diffuse alveolar injury and capillary endothelial damage, is a common clinical syndrome with high a mortality rate (Wang et al., 2019d). lncRNA Xist (Wang et al., 2019d), which acts as a ceRNA to negatively upregulate IRF2 (interferon regulatory factor 2) expression to sponge miR-204, significantly decreases the PaO2/FiO2 ratio and aggravates lipopolysaccharide-induced ARDS in mice by regulating the miR-204/IRF2 axis.

In the parthenogenetic development of pigs, silencing lncRNA Xist remarkedly increased the total blastocyst cell number but did not influence the rate of embryo cleavage and blastocyst formation compared with the control group (Chen et al., 2019e). This study suggested that lncRNA Xist may play a role in a new approach for improving the quality of porcine parthenogenetic embryos. lncRNA Xist (Zhang et al., 2019b) facilitates cells development in somatic cells by TALE-based designer transcriptional factor, and regulates embryonic stem cells’ fates (Chelmicki et al., 2014; An et al., 2020). lncRNA Xist promotes hair follicle regeneration in Dermal papilla cells via regulating miR-424/Shh axis to activate hedgehog signaling (Lin B.J. et al., 2020), and regulates HT cell proliferation and invasion in human trophoblast (HT) cells via miR-144/Titin axis by activating the downstream MAPK and MMPs pathway (Yu N.H. et al., 2017). In polycystic ovary syndrome (PCOS), lncRNA Xist is correlated with adverse pregnancy outcomes (Liu et al., 2020c). In addition, these signaling pathways, which include lncRNA Xist/miR-203-3p/ZFPM2 (Niu et al., 2020), lncRNA Xist/let-7c-5p/STAT3 (Wang et al., 2020d), and lncRNA Xist/miR-320/NOD2 (Xu X.H. et al., 2018), have been identified as a ceRNA regulatory network and participated in osteoblast development and ox-LDL (oxidative low-density lipoprotein)-induced endothelial cells injury.

A previous study reported that lncRNA Xist contributed to human skin fibroblasts by serving as a miRNA sponge. However, lncRNA Xist (Guo et al., 2018; Cao and Feng, 2019) regulates these processes containing skin fibroblasts proliferation, migration, and ECM (extracellular matrix) synthesis after thermal injury by sponging miRNAs (miR-29a and 29b-3p) to promote the expression of target genes (LIN28A and COL1A1). Additionally, lncRNA Xist/miR-181a/COL4A1 axis (Tian R. et al., 2020) is involved in the development and progression of keratoconus using transcriptome RNA-seq data assay. All in all, these results demonstrated that lncRNA Xist plays a pivotal function in non-cancer diseases.

## Discussion and Perspectives

**FIGURE 3:**
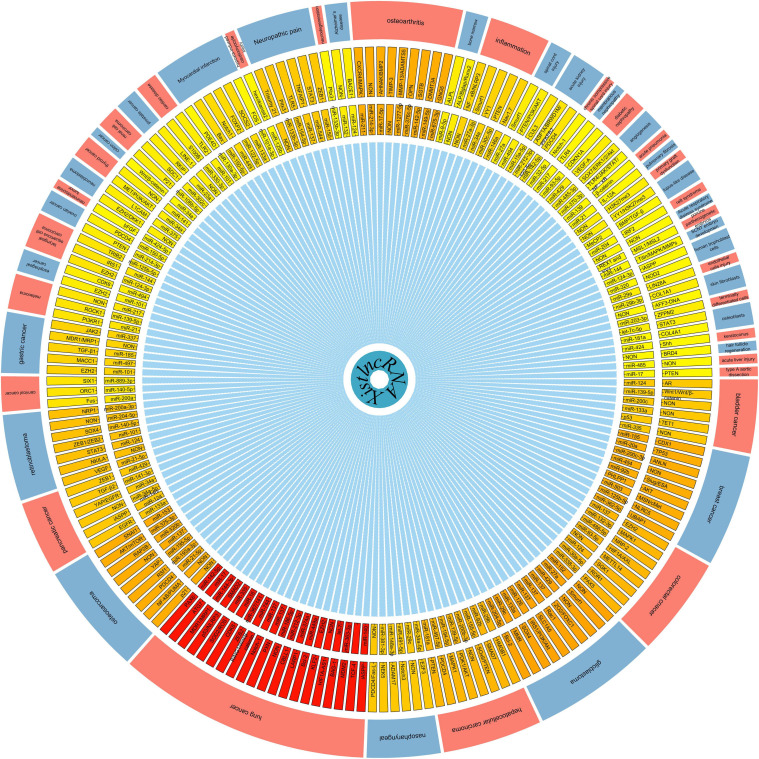
Overview of the regulatory network of lncRNA Xist involved in mammalian diseases and cells. NON, The downstream target Unclear.

LncRNA Xist, which is conserved among eutherians (human, Rat, mouse, cow, dog, and elephant) but not non-eutherian vertebrates, is an important initiator of the process of XCI in eutherian mammals (Brockdorff et al., 1991; Duret et al., 2006; Galupa et al., 2020). lncRNA Xist is produced by Xist gene and is up-regulated from the Xi chromosome during the XCI process, and recruits protein complexes to reprogram chromosomes [such as H3K27me3 and H2AK119ub trimethylation (Postlmayr et al., 2020)]. In addition to its original XCI functions, numerous studies (Chaligne and Heard, 2014; Dey et al., 2014; Schmitz et al., 2016; Yang Z. et al., 2018; Cheng J.T. et al., 2019; Yan et al., 2019) have also indicated that lncRNA Xist is related to the pathogenic process of multiple diseases by regulating of cell migration, invasion, apoptosis, differentiation, proliferation, and drug resistance. Further investigation of lncRNA, which is considered to function as a miRNA or gene regulator, may aid in addressing disease etiology, such as lung cancer, breast cancer, glioblastoma, osteoarthritis, neuropathic pain, heart disease, and inflammation (Tables 1, 2). By summarizing current knowledge, we noticed that the regulatory network of lncRNA Xist in the majority of biological processions varies considerably. However, lncRNA Xist appears to regulate these processes primarily by interacting with miRNAs to positively facilitate downstream target gene expression (Figure 3). Further studies showed that the regulatory network of lncRNA Xist participated in various signaling pathways, such as TGF-beta signaling pathway, PIK3/AKT signaling pathway, Wnt/β-catenin signaling pathway, FOXO signaling pathway, NF-kB signaling pathway, mTOR signaling pathway, MAPK signaling pathway, Toll-like receptor signaling pathway, JAK-STAT signaling pathway, T cell receptor signaling pathway, and B cell receptor signaling pathway (Tables 1, 2). Although lncRNA Xist taking part in these signaling pathway functions has rarely been demonstrated, there is no reason to believe that the unexplored functions of lncRNA Xist will not be understanded in these ways. These mechanisms of lncRNA Xist action in diseases can indirectly and directly provide recommendations for future research, and more functions of lncRNA Xist can be confirmed.

In theory, the genes lncRNA Xist over-expresses and silences are numerous. But over-expression of lncRNA Xist, which is a 15–17 kb RNA polymerase II transcript that is both spliced and polyadenylated (Brockdorff, 2019), is different when using plasmids. By contrast, lncRNA Xist may be inhibited using small molecule inhibitors that block specific binding sites (Matoba et al., 2011). Based on the above reasons, understanding of the function of lncRNA Xist is in its infancy for various diseases and cells. With the developing genome editing technology (Du and Qi, 2016; Chen et al., 2019b; Yi and Li, 2020), CRISPR/Cas9 system is emerging as a powerful tool for sequence-specific control of lncRNA Xist expression in mammalian cells. Recently, numerous studies (Yue and Ogawa, 2018; Colognori et al., 2019; Waśko et al., 2019; Deng et al., 2020) have indicated that CRISPR/Cas9 system is useful for studying lncRNA Xist function and related ceRNA regulatory networks. By combining other future technologies, the function and mechanism of lncRNA Xist will certainly be found. Investigation of lncRNA Xist in virous cells may uncover numerous novel therapeutic approaches for disease treatment in the future. At the same time, it might result in a better understanding of how lncRNA Xist contributes to the XCI and diseases in mammals, potentially opening new avenues for research and therapeutic manipulation of these diseases.

## Author Contributions

DZ, WW, and LZ: manuscript design. WW, JM, and XW: literature collection and summary. WW and LZ: drafting of the manuscript. WW, LM, and CL: figure drawing. XQ, WW, DZ, and LZ: revising of the manuscript. All authors have read and approved the final submitted manuscript.

## Conflict of Interest

The authors declare that the research was conducted in the absence of any commercial or financial relationships that could be construed as a potential conflict of interest.

## Glossary

lncRNAs, Long non-coding RNAs; lncRNA Xist, Long non-coding RNA X-inactive specific transcript; XIC, X chromosome inactivation center; ceRNA, competing endogenous RNA; XCI, X-chromosome inactivation; Xa, active X chromosome; Xi, inactive X chromosome; Ftx, Ftx transcript, Xist regulator; Jpx, Jpx transcript, Xist activator; Tsix, Tsix transcript, Xist antisense RNA; OCT4, also known as Pou5f1; CTCF, CCCTC-binding factor; PRC1 and PRC2, Polycomb repressive complexes (PRCs) -PRC1 and -PRC2; RNA Pol II, RNA polymerase II; hnRNPU (also known as SAF-A), heterogeneous nuclear protein U; ATRX, ATRX chromatin remodeler; hnRNPK, Heterogeneous nuclear ribonucleoprotein K; SHARP, SPEN family transcriptional repressor; HDAC3, histone deacetylase 3; LBR, lamin B receptor; Airn, antisense of IGF2R non-protein coding RNA; Kcnq1ot1, KCNQ1 opposite strand/antisense transcript 1; RBM15, RNA binding motif protein 15; WTAP, WT1 associated protein; U1 snRNP, U1 small nuclear ribonucleoprotein; Rsx, RNA-on-the-silent X; NANOG, Nanog homeobox; SOX2, SRY-box transcription factor 2; PRDM14, PR/SET domain 14; REX1, ZFP42 zinc finger protein; RNF12, RING-H2 finger protein; CdK8, cyclin dependent kinase 8; AR, Androgen Receptor; BCSC, bladder cancer stem cell; PD, Platycodin D; BRCA1, Breast Cancer 1 protein; EMT, epithelial mesenchymal transition; CDX1, caudal-type homeobox 1; TNBC, Triple-negative breast cancer; ESA, epithelium-specific antigen; RELA, NF-κB subunit; BCSCs, Breast cancer stem cells; PHLPP1, PH domain and leucine-rich repeat phosphatase 1; ZEB1, zinc finger E-box binding homeobox 1; EZH2, enhancer of zeste homolog 2; MAPK1, mitogen-activated protein kinase 1; NRP-2, neuropilin-2; HIF-1A, hypoxia inducible factor 1, alpha subunit; METTL14, methyltransferase-like14; YTHDF2, YTHDF proteins 2; SGK1, serum and glucocorticoid-inducible kinase 1; ROR1, receptor-tyrosine-kinase-like orphan receptor 1; Smurf1, smad ubiquitination regulatory factor 1; FOXC1, forkhead box C1; ZO-2, zonula occludens 2; Rac1, Rac family small GTPase 1; ASCT2 and SLC1A5, alanine-, serine-, and cysteine-preferring transporter 2; PI3K, phosphatidylinositol-4,5-bisphosphate 3-kinase; IRS1, insulin receptor substrate 1; SOX4, Y-related high-mobility group box 4; TMZ, Temozolomide; MMR, DNA mismatch repair; HBV, hepatitis B virus; HCV, hepatitis C virus; HMGB1, high-mobility group box-1; PDK1, pyruvate dehydrogenase kinase 1; TNM, tumor-node-metastasis; PDCD4, programmed cell death 4; PTEN, phosphatase and tensin homolog; E2F3, E2F transcription factor 3; ADAM17, a disintegrin and metalloproteinase 17; NEK5, NIMA related kinase 5; TAM, tumor-associated macrophage; TCF-4T, T-cell-specific transcription factor 4; MDM2, mouse double minute clone 2; BAG-1, bcl-2 associated athanogene-1; NSCLC, Non-small cell lung carcinoma; LARP1, La-related protein 1; CBLL1, Casitas B-lineage proto-oncogene like 1; TGF-β1, Transforming growth factor β; ZEB2, zinc finger E-box binding homeobox 2; ATG7, autophagy associated gene 7; PUMA, p53 upregulated modulator of apoptosis; NF-kB, nuclear factor-kappa B; YAP, Hippo/yes-associated protein; EGFR, epidermal growth factor receptor; iASPP, inhibitor of apoptosis-stimulating protein of p53; CDK1, Cyclin-dependent kinase 1; VEGF, vascular endothelial growth factor; STAT3, signal transducer and activator of transcription 3; MACC1, MET transcriptional regulator; MDR1, multidrug resistance gene 1; MRP1, multi-drug resistance protein 1; bFGF, basic fibroblast growth factor; SDC1:Syndecan-1; TGCT, testicular germ cell tumor; CAD, coronary artery disease; MI, myocardial infarction; HF, heart failure; TLR2, toll-like receptor 2; PDE4D, Phosphodiesterase 4D; CB2R, cannabinoid receptor type II; LR5, toll-like receptor 5; NFAIP1, tumor necrosis factor alpha-induced protein 1; CCI, chronic constriction injury; AD, Alzheimer’s disease; OA, Osteoarthritis; PDLSCs, periodontal ligament stem cells; OP, osteoporosis; ALP, alkaline phosphatase; MMP-13, matrix metalloproteinase 13; ADAMTS5, ADAM metallopeptidase with thrombospondin type 1 motif 5; OPN, overexpression of osteopontin; TLRS, Toll−like receptors; CRPS, Complex regional pain syndrome; YY1, Yin-Yang 1; SCI, Spinal cord injury; CCSCI, Compressive Spinal Cord Injury; CDKN1A, cyclin-dependent kinase inhibitor 1A; PFOS, Perfluorooctane sulfonate; SOX7, SRY-box 7; PGD, Primary graft dysfunction; NET, neutrophil extracellular trap; NOD2, Nucleotide-Binding Oligomerization Domain 2; COL1A1, collagen 1 alpha 1.
